# Supplementary material for: Content-rich biological network constructed by mining PubMed abstracts
Source: BMC Bioinformatics. 2004 Oct 8;5:147. doi: 10.1186/1471-2105-5-147 (PMC528731; doi:10.1186/1471-2105-5-147)
Supplement: Additional File 5 — The original Chilibot query results of the term "long-term potentiation (LTP)" and 22 other terms, limiting the latest references analyzed to the years 1990, 1995, 2000, and 2004. [file 1471-2105-5-147-S5.bz2 › chilibotAdditionalFile5/ltp2000/html/ERK_CAMKII.html]

 


 **ERK** and **CAMKII** 
  
Found 3 abstracts in PubMed,  **3 abstracts were retrieved and analyzed**.  


---

 Search Google  |
 PDF files only 
|  EDU domain only 

---

**Interactive relationship** (e.g. stimulation, inhibition, etc)

- the effects of  **ERK**  inhibition on migration may be mediated through its ability to inhibit  **CamKII**  activity.  Ref: 9925373 J Mol Cell Cardiol, 1998
- Although KN62 did not affect ERK2 activity in response to PDGF, PD98059 markedly inhibited PDGF stimulated  **CamKII**  activity, suggesting that activation of  **CamKII**  by PDGF was dependent on  **ERK**  activity.  Ref: 9925373 J Mol Cell Cardiol, 1998

**Parallel relationship** (e.g. studied together, co-existance, homology, etc.)

- Phosphorylation consensus sites for various kinases exist within the sequence of the potassium channel subunit Kv4.2, including consensus sites for extracellular signal regulated kinase mitogen activated protein kinase  **ERK**  MAPK, protein kinase A PKA, protein kinase C PKC, and calcium calmodulin dependent kinase II  **CaMKII** , and kinase assays have shown that particular amino acids of the consensus sites are bonafide phosphorylation sites in vitro.  Ref: 11040264 Learn MemLearn Mem, 1998
